# Editorial: Genetic and environmental interactions in oral disease: advancing diagnostic and therapeutic strategies

**DOI:** 10.3389/fmed.2025.1669468

**Published:** 2025-08-12

**Authors:** Andrea Ballini, Nicola Silvestris, Flavio Pisani, Danila De Vito

**Affiliations:** ^1^Department of Life Science, Health and Health Professions, Link Campus University, Rome, Italy; ^2^Medical Oncology Unit, Department of Human Pathology “G. Barresi”, University of Messina, Messina, Italy; ^3^School of Medicine and Dentistry, University of Central Lancashire, Preston, United Kingdom; ^4^School of Medicine, University of Bari Aldo Moro, Bari, Italy

**Keywords:** precision medicine, health promotion, translational research, preventive medicine, medicine and dentistry, oral health and clinical microbiology, biochemistry, clinical biochemistry and molecular clinical biology

Pathology in dentistry is a critical field that delves into the study of diseases affecting the oral cavity and its surrounding structures (1–4). This area of research is pivotal for identifying, diagnosing, and understanding a wide array of oral health conditions, such as infections, inflammations, developmental abnormalities, and neoplastic growths (2–4). The exploration of these conditions is essential not only for dental health but also for overall health, as oral diseases can have systemic implications. Despite significant advancements, there remain gaps in our understanding of the underlying causes, mechanisms, and progression of these diseases. Recent studies have highlighted the importance of precision medicine in addressing these gaps, yet there is a need for more comprehensive research that integrates clinical and pathological aspects to improve patient care (5–7). The ongoing debate in the field revolves around the integration of genetic and environmental factors in oral disease etiology, necessitating further investigation to enhance diagnostic and therapeutic strategies (8–11).

The articles in this topic highlight the advances in the field of oral diseases by promoting research that covers both clinical and pathological aspects. The primary objective was to enhance the standard of care for patients by integrating precision medicine approaches into dentistry.

In his work, Petruzzi, describe a simple technique for assessing the presence of blisters and vesicles on the oral mucous membranes using the air syringe, which is a standard component of every dental unit.

Specific questions to be addressed include the identification of genetic risk factors for oral diseases, the interaction between genetic and environmental factors, and the causal links between oral and systemic diseases. By fostering interdisciplinary and collaborative research, this topic seeks to bridge existing knowledge gaps and contribute to the development of innovative diagnostic and therapeutic solutions.

In their study Padmanabhan et al. aims to evaluate salivary alpha-amylase levels in children diagnosed with Early Childhood Caries (ECC) and Rampant Caries (RC), and to compare these levels with those found in children without ECC or RC. Additionally, it investigates the relationship between salivary alpha-amylase concentrations and increased caries activity in children affected by ECC or RC.

Oral potentially malignant disorders (OPMDs), including conventional leukoplakia (OL) and proliferative verrucous leukoplakia (PVL), have distinct risks of progression to oral squamous cell carcinoma (OSCC).

Intini et al., aimed to analyze and compare the oral microbiota in patients with OL, PVL, and OSCC using 16S rRNA gene sequencing of saliva samples to identify microbial signatures associated with disease progression and to uncover potential biomarkers that would justify an aggressive treatment of OPMDs.

Previous studies have suggested that genetic polymorphisms may influence the clinical manifestations of temporomandibular disorder (TMD). Accordingly, Baratto et al. study aimed to examine the association between polymorphisms in the *Dopamine Receptor D2* (*DRD2*) and *Ankyrin Repeat and Kinase Domain Containing 1* (*ANKK1*) genes and the oral health-related quality of life in male patients with TMD.

Finally, in a review, Myo et al. explores the role of Msx1 in cleft palate, offering a detailed overview of its functions and the molecular mechanisms by which it regulates palatal development. We highlight recent research findings, including studies on *Msx1* mutations, signaling pathways, and gene–environment interactions, to shed light on the complex association between *Msx1* and cleft palate. Furthermore, ongoing advances in research may position *Msx1* as a key target for the development of innovative therapeutic approaches to craniofacial disorders.

In conclusion, all the articles dispensed an update on the latest evidence regarding the Genetic and Environmental Interactions in Oral Disease, highlighting the advancing Diagnostic and Therapeutic Strategies.

## References

[1] Grigolato R Bizzoca ME Calabrese L Leuci S Mignogna MD Lo Muzio L. Leukoplakia and immunology: new chemoprevention landscapes? Int J Mol Sci. (2020) 21:6874. 10.3390/ijms21186874 32961682 PMC7555729

[2] Sharma H Sen S Lo Muzio L Mariggiò A Singh N. Antisense-mediated downregulation of anti-apoptotic proteins induces apoptosis and sensitizes head and neck squamous cell carcinoma cells to chemotherapy. Cancer Biol Ther. (2005) 4:720–7. 10.4161/cbt.4.7.1783 15917659

[3] Vieira E. Silva FF, Caponio VCA, Ballini A, Chamorro-Petronacci CM, Lourenzo-Pouso AI, García-García A, Di Domenico M, Suaréz-Peñaranda JM, Pérez-Sayáns M, Padín-Iruegas ME. Smac/DIABLO protein acts as an independent prognostic factor in oral squamous cell carcinoma. Sci Rep. (2024) 14:30065. 10.1038/s41598-024-76962-1 39627250 PMC11614858

[4] Dioguardi M Musella G Bizzoca ME Sovereto D Guerra C Laterza P. The prognostic role of miR-375 in head and neck squamous cell carcinoma: a systematic review, meta-analysis, and trial sequential analysis. Int J Mol Sci. (2025) 26:2183. 10.3390/ijms26052183 40076805 PMC11900050

[5] Mauceri R Coppini M Pérez-Sayáns M Toro C Vitagliano R Colella G. Challenges in the diagnosis of oral squamous cell carcinoma mimicking medication-related osteonecrosis of the jaws: a multi-hospital-based case series. Oral Oncol. (2024) 151:106689. 10.1016/j.oraloncology.2024.106689 38503259

[6] Dioguardi M Bizzoca ME Cantore S Caloro GA Musella G Mastrangelo F. Impact of cerebrovascular stroke on inflammatory periodontal indices: a systematic review with meta-analysis and trial sequential analysis of case-control studies. Front Oral Health. (2024) 5:1473744 (Erratum in: Front Oral Health. 2025; 6:1580261). 10.3389/froh.2025.1580261 39512558 PMC11540815

[7] Foti C Romita P Rigano L Zimerson E Sicilia M Ballini A. Isobornyl acrylate: an impurity in alkyl glucosides. Cutan Ocul Toxicol. (2016) 35:115–9. 10.3109/15569527.2015.1055495 26095233

[8] Crincoli V Cazzolla AP Di Comite M Lo Muzio L Ciavarella D Dioguardi M. Evaluation of vitamin D (25OHD), bone alkaline phosphatase (BALP), serum calcium, serum phosphorus, ionized calcium in patients with mandibular third molar impaction. An observational study. Nutrients. (2021) 13:1938. 10.3390/nu13061938 34200107 PMC8228145

[9] Musella G Coppini M França Vieira E Silva F Campisi G Pérez-Sayáns M Caponio VCA. Tumor-stroma ratio in head and neck squamous cell carcinoma: a systematic review and meta-analysis. Oral Dis. (2025). 10.1111/odi.15319. [Epub ahead of print]. PMC12423486 40127161

[10] Ballini A Cantore S Fatone L Montenegro V De Vito D Pettini F. Transmission of nonviral sexually transmitted infections and oral sex. J Sex Med. (2012) 9:372–84. 10.1111/j.1743-6109.2011.02515.x 22023797

[11] Musella G Liguori S Cantile T Adamo D Coppola N Canfora F. Knowledge, attitude and perception of Italian dental students toward HPV-related oropharyngeal cancer and vaccination: a cross-sectional study. BMC Oral Health. (2024) 24:1213. 10.1186/s12903-024-04998-w 39402502 PMC11472497

